# Unknown Enemy and Psychopathological Responses: A Cross-Sectional Nationwide Study Assessing the Knowledge About COVID-19

**DOI:** 10.3389/fpsyt.2021.704558

**Published:** 2021-08-11

**Authors:** Julian Maciaszek, Marta Lenart, Błazej Misiak, Jolanta Grzebieluch, Paweł Gawłowski, Marta Ciułkowicz, Dorota Łuc, Dorota Szcześniak, Joanna Rymaszewska

**Affiliations:** ^1^Department of Psychiatry, Wroclaw Medical University, Wroclaw, Poland; ^2^Department of Public Health, Wroclaw Medical University, Wroclaw, Poland; ^3^Department of Emergency Medical Service, Wroclaw Medical University, Wroclaw, Poland; ^4^Practice of Family Doctors M.V. Domanscy, Wroclaw, Poland

**Keywords:** COVID-19, SARS-CoV-2, pandemic, disease knowledge, mental health, anxiety, somatic symptoms, infodemic

## Abstract

There is evidence that a lack of appropriate knowledge regarding global changes might be associated with various psychopathological responses. In this study, we tested the hypothesis that knowledge about COVID-19 correlates with the severity of psychopathological symptoms as measured by standardized questionnaires. The questionnaires were obtained using the Computer Assisted Web Interviews (CAWI) method during the second wave of the COVID-19 pandemic in Poland using the original COVID-19 knowledge questionnaire and the General Health Questionnaire-28 (GHQ-28). A series of bivariate tests and linear regression analyses were performed with a *p* < 0.05. All analyses were performed in Statistica 13.3. We enrolled 1,002 respondents. The rate of correct answers in the original questionnaire ranged from 44.6 to 84.1%, and the average was 60.1%. Four hundred and twenty participants (42%) met the criterion for the presence of relevant psychopathological symptoms. A significant negative correlation was found between the number of points obtained in the COVID-19 knowledge questionnaire and the GHQ-28 scores, both in relation to the total score and all its subscales. The following factors in the linear regression model were correlated with severity of somatic symptoms: knowledge about the COVID-19 pandemic (*B* = −0.12, *P* = 0.000), sex (*B* = 0.12, *P* = 0.000), use of psychiatric or psychological care (*B* = 0.20, *P* < 0.000) and chronic diseases (*B* = 0.09, *P* = 0.002). In this study, we observed a negative correlation between the knowledge about the COVID-19 pandemic and the severity of psychopathological symptoms. The results clearly indicate that the complexity of the global problem of the current pandemic is related to the development of psychopathological symptoms. However, longitudinal studies are needed to identify the direction of causality.

## Introduction

A new type of Coronaviruses Severe Acute Coronavirus Disease-2 (SARS-CoV-2) causing Coronavirus Disease 2019 (COVID-19) has been reported for the first time in China in December 2019 (1), and on March 11, 2020, the World Health Organization (WHO) announced pandemic (2). In the initial period of the pandemic, analyzes performed by WHO Collaborating Centre for Infectious Disease Modeling predicted that the effects of the SARS-COV-2 pandemic would be comparable to the 1918 flu pandemic (3), which had resulted in 50 million deaths and 28 years mean death age. At the time of writing this article, we are witnessing a second wave of the virus epidemic and, according to the recommendations introduced by the WHO regarding the principles of reporting deaths (4), so far, approximately 1.7 million people have died due to COVID-19 (5). Depending on the country, from 77.3 to 95.5% of them were above 65 years old (6). The Infection Fatality Rate was found to range from 0.3 to 0.6% (7, 8). Most countries around the world with a few exceptions such as Sweden (9) and Belarus (10) according to WHO's recommendations in order to flatten the disease curve have introduced far-reaching restrictions (11, 12), which lead to serious economic crisis (13). During pandemic, there has been a significant reduction in the total number of hospitalizations and elective procedures (14, 15), including hospitalizations due to acute coronary syndrome (16, 17) and total reduction of cancer surgeries (18). Consequently, it was observed an increase in the total number of deaths compared to previous years, regardless of the impact of deaths caused by COVID-19 (19).

Some researchers point out that in addition to somatic symptoms of COVID-19, there are also neuropsychiatric manifestations, but more research is still needed (20, 21). Sonderskov et al. (22) in a study using WHO-5 well-being scale (23) found that there is a significantly lower well-being during the pandemic period compared to the pre-COVID-19 pandemic period in general society. In our previous nationwide cross-sectional study, we have reported that more than 50% of respondents (independently of profession) experienced clinically significant psychiatric symptoms during the first weave of pandemic (24). According to the meta-analysis by Bueno-Notivol et al. (25), the pooled prevalence of depression in society during the COVID-19 pandemic is estimated at 25%—approximately seven times greater compared to the average prevalence of depression before the pandemic estimated at 3.44%. Social isolation and being in quarantine are also known to trigger serious consequences such as post-traumatic stress symptoms, anxiety, depression, fear, anger, confusion and a reduction in the quality of life (26, 27).

A huge information chaos and an increasing number of fake news present in the media, known as infodemic, have been observed in the public space (28, 29) leading to growing anxiety related to coronavirus (30, 31). As reported by Dubey et al. (32) during the pandemic the negative impact of the disease itself on mental health was multiplied as a result of nationwide lockdowns and quarantines, as well as the phenomenon of infodemic, leading in the long term to the occurrence of acute panic, anxiety, obsessive behaviors, compulsive hoarding, paranoia, depression and post-traumatic stress disorder. In the case of the Ebola virus epidemic (33) the notably increased frequency of information had a significant impact on the escalation of public concerns, causing an increased sense of threat, anxiety and uncertainty about the future. In the case of the swine-origin influenza A (H1N1) epidemic, a negative impact of social media was observed in the form of immediate flooding of users with information, leading to growing fear and anxiety, regardless of the real threat (34). The impact on mental health of the rapidly spreading unverified and extreme information called “fake news” cannot be underestimated (35). According to Roy D. et al. (36) study, almost half of the respondents felt panic after reading electronic and printed media reports on COVID-19. As reported by Gao et al. (37), in the initial period of the pandemic, increased exposure to social media was associated with a higher level of anxiety. This information is closely related to the report of Nekliudov et al. (38), based on a nationwide study reporting that time spent tracking COVID-19 reports correlated with a higher level of anxiety.

Appropriate knowledge in the society regarding the scale of the epidemiological threat facilitates the application of appropriate preventive measures that correspond to the real degree of threat, and thus, enable the prevention of the epidemic spread (39). According to Thomas et al. (40) mainstream broadcasting media is a tool that can significantly expand knowledge about epidemiological threats by influencing pro-health behavior, but at the same time, it can be used to manipulate facts, giving an incomplete picture of the situation and, as a result, negatively affect public health. With regard to the study performed in 2016 during H1N1 virus epidemic, a significant proportion of the respondents presented insufficient knowledge about the severity and preventive measures (39). Similar results were obtained in a study conducted among secondary school students, where most participants had insufficient knowledge and a negative attitude toward the Ebola virus epidemic in 2015 (41). In the review of studies assessing knowledge about COVID-19, Puspitasari et al. (42) point to a generally high level of knowledge that is comparable to both medical professionals and non-medical professionals. A study by Zhong et al. (43) showed that greater knowledge about COVID-19 was associated with an optimistic attitude and the use of preventive practices. According to Lei et al. (44) higher self-evaluated level of knowledge correlated with higher severity of depressive and anxiety symptoms. Alzoubi et al. (45) highlighted in his study that social media among students is the main source of knowledge about COVID-19. At the time writing this article there are no published studies evaluating the relationship between COVID-19 knowledge and mental health assessed using standardized questionnaires. In order to bridge this research gap, we investigated whether the knowledge about COVID-19 is related to the severity of psychopathological symptoms. We hypothesized that lower level of knowledge about the COVID-19 is related to higher severity of psychopathological symptoms.

## Materials and Methods

The data were collected through an online survey conducted from September 26, 2020 to October 27, 2020, i.e., during the development of the second wave of the SARS-CoV-2 pandemic in Poland. At the time of data collection, a significant increase in the number of positive test results was observed, initiating subsequent stages of restrictions, including the order to cover the mouth and nose in public spaces, introduced from October 10, 2020 (46). The questionnaires were obtained using the Computer Assisted Web Interviews (CAWI) method, which is currently one of the most popular and fastest growing survey methods. Thanks to the feeling of anonymity and the opportunity to participate in the survey at a time convenient for the respondent, it allows to collect more reliable data. The manuscript was formulated based on STROBE Statement—cross-sectional reporting checklist (47).

The study was partly community based and partly open to the public. Participants over the age of 18 were invited to complete an anonymous Google Forms survey distributed via social media (Facebook, WhatsApp), and information about the survey was also posted on the website of the Department of Psychiatry of the Wroclaw Medical University. In the case of people willing to complete the survey who do not use social media, the survey was also distributed 54 times at the request of interested persons via e-mail. All participants signed consent to participate in the survey. The study was approved by the Ethics Committee of the Medical University of Wroclaw (Poland, no 188/2020) and performed in accordance with the principles of the Declaration of Helsinki. A priori analysis performed using G^*^ Power software (48) revealed that to detect a correlation with *r* = 0.01 and power of 0.95, the calculated sample size was 595. Due to the potential non-response, questionnaires were sent to more participants. The final sample size was 1,002.

The survey consisted of three sections: sociodemographic section, the original questionnaire of knowledge about COVID-19 and the General Health Questionnaire-28 (GHQ-28). The first section contained information on sex, age, education, place of residence, occupation, somatic and mental disorders. The original COVID-19 knowledge questionnaire consisted of 10 single-choice questions, where the participant had to choose one answer from among three available. The questions were constructed based on definitions and information provided by WHO (49), reports of the Central Statistical Office (50), the Chief Sanitary Inspectorate (51) and the Ministry of Health (52). For each correct answer, participants received 1 point, for the incorrect answer-−0 points. The points ranged from 0 to 10, with more points indicating better knowledge about COVID-19. Question number 1 regarded the current definition of a pandemic, questions 2,3,4,6 concerned the virulence and course of SARS-CoV-2 infection, questions 5,7,8 concerned the measurable effects of the pandemic, and questions 9 and 10 regarded knowledge of personal protective equipment (Supplementary Table 1). The number of correct answers was included as the measure of knowledge. To ensure the reliability of the items, the COVID-19 knowledge questionnaire was pre-tested in 160 respondents. The Cronbach's alpha was 0.724 in the pretested subgroup of participants. The Cronbach's alpha in the total sample was 0.716, indicating acceptable internal consistency.

The GHQ-28 is a 28-item questionnaire used for general identification of minor mental disorders in the general population and is divided into four subscales. These are: somatic symptoms (items 1, 3, 4, 8, 12, 14, 16), anxiety and insomnia (items 2, 7, 9, 13, 15, 17, 18), social dysfunctions (items 5, 10, 11, 25, 26, 27, 28) and severe depression (items 6, 19, 20, 21, 22, 23, 24) (53, 54). The answers are scored on a 4-point Likert scale (0-not at all, 1-no more than usual, 2-rather more than usual, and 3-much more than usual). The total score ranges from 0 to 84, with higher scores corresponding to higher levels of disorders. In the study we used the Polish adaptation of the questionnaire. The cut-off point for clinical relevance was set at 24 points, as described by Makowska and Merecz (53) according to the previous validation for the target population of the study.

The following procedure was used: anonymous responses received via Google Forms were identified by code numbers, checked for completeness and submitted for further analysis.

Only fully completed questionnaires were used for statistical analysis. The Mann-Whitney *U*-test or *t*-test, as appropriate, were used to compare respondents with respect to continuous variables. The chi-square test was used to test differences in categorical variables. The Spearman rank correlation coefficients were used to test associations between continuous variables. *Post hoc* power analysis of two independent correlations were performed using G^*^ Power software (48). Additionally, linear regression analysis with backward stepwise selection was performed. The GHQ-28 scores were included as a dependent variable. Independent variables were selected after a series of bivariate tests. More specifically, variables associated with either the number of correct answers on the COVID-19 knowledge questionnaire or the GHQ-28 scores were included as independent variables. The results were considered significant if the *p*-value was less than 0.05. All analyses were performed in Statistica 13.3.

## Results

### General Characteristics

The general characteristics of the sample are presented in Table 1. The questionnaire was completed by a total of 1,002 participants. Among the respondents, the mean age was 38 years, 750 out of 1,002 participants (75%) were women. Seven hundred and thirty four participants (73%) lived in the city, 761 had higher education level (76%), 268 (27%) worked in a health care service, 214 (21%) suffered from chronic diseases and 175 (18%) received psychiatric or psychological care. The mean score obtained in the COVID-19 knowledge questionnaire was 6.0 (standard deviation [*SD*]: 2.1, range: 0–10). The rate of correct answers to specific questions ranged from 44.6 to 84.1% and the average was 60.1% (Supplementary Table 1). Four hundred and twenty out of 1,002 participants (42%) met the criterion for the presence of relevant psychopathological symptoms (the GHQ-28 total score > 24). Mean score in the GHQ-28 was 23.0 (*SD*: 12.9, range: 1–75).

**Table 1:** General characteristics of the study sample.

**Variable**	***N* (%) or mean ± standard deviation**
Age	38.4 ± 14.6
Sex (female)	750 (74.9%)
Place of residence (rural)	268 (26.8%)
Education level (higher education)	761 (75.9%)
Occupation (medical profession)	268 (26.8%)
Chronic diseases (yes)	214 (21.4%)
Psychiatric or psychological care (yes)	175 (17.5%)
GHQ-28 total	22.86 ± 12.9
GHQ-28 severe depression	2.96 ± 3.8
GHQ-28 social dysfunction	7.66 ± 2.9
GHQ-28 anxiety and insomnia	6.58 ± 4.7
GHQ-28 somatic symptoms	5.66 ± 3.8
Knowledge about COVID-19: number of correct answers	6.00 ± 2.1

*Data expressed as n (%) or mean (SD)*.

### Bivariate Comparisons

Significant differences were observed in the GHQ-28 total results depending on sex, age, level of education, presence of chronic diseases and the use of psychiatric and psychological care (Supplementary Table 2). Knowledge scores differed significantly depending on sex, education level, and the use of psychiatric or psychological care (Supplementary Table 3). A significant negative correlation was found between the number of points obtained in the COVID-19 knowledge questionnaire and the GHQ-28 scores, both in relation to the total score and all its subscales (Table 2). *Post hoc* determined power analysis of correlations ranged from 0.643 to 0.973.

**Table 2:** Correlations of the total number of points obtained in the COVID-19 knowledge questionnaire and the severity of psychopathological symptoms.

**Variable**	***r*-Value**	***P*-Value**	**Power (1- β err. prob.)**
GHQ-28 total	0.15	** <0.000**	0.956
GHQ-28 severe depression	0.10	**0.001**	0.722
GHQ-28 social dysfunction	0.09	**0.004**	0.643
GHQ-28 anxiety and insomnia	0.14	** <0.000**	0.931
GHQ-28 somatic symptoms	0.16	** <0.000**	0.973

*Significant effects (p< 0.05) are marked in bold*.

### Linear Regression Analysis

Table 3 presents the results of the linear regression analysis testing for factors significantly related to the GHQ-28 total score. Male sex, higher education, a negative history of psychiatric or psychological care were associated with lower severity of psychopathological symptoms showed by the lower total GHQ-28 scores. The results of the linear regression analysis indicate that the following factors were associated with a lower severity of the GHQ-28 severe depression subscale: age, higher education, no use of psychiatric or psychological care. Factors such as older age and a negative history of using psychiatric care were correlated with lower severity of symptoms in GHQ-28 social dysfunction scale. According to results of linear regression regarding to the GHQ-28 anxiety and insomnia subscale, male sex, higher education level and no use of psychiatric or psychological care were associated with a lover severity of symptoms. The following factors in the linear regression model were associated with less severity of the somatic symptoms subscale: greater knowledge about the COVID-19 pandemic, male sex, lack of chronic diseases, and no use of psychiatric or psychological care.

**Table 3:** Factors related to the GHQ-28 score (results of linear regression analysis).

	**GHQ-28 total**	**GHQ-28 severe depression**	**GHQ social dysfunction**	**GHQ-28 anxiety and insomnia**	**GHQ-28 somatic symptoms**	***VIF***
Knowledge about COVID-19: number of correct answers	*B* = −0.09, *P* = 0.003	*B* = −0.05, *P* = 0.58	*B* = −0.07, *p* < 0.15	*B* = −0.09, *P* = 0.004	***B*****=** –**0.12,** ***P*** **=** **0.000**	1.06
Age	*B* = −0.07, *P* = 0.01	***B*****=** –**0.14,** ***P*****<** **0.000**	***B*****=** –**0.12**, ***P*** **<** **0.000**	*B* = −0.01, *P* = 0.67	*B* = −0.05, *P* = 0.07	1.12
Sex	***B*** **=** **0.11,** ***P*** **<** **0.000**	*B* = 0.02, *P* = 0.33	*B* = 0.02, *P* = 0.35	***B*** **=** **0.13,** ***P*** **<** **0.000**	***B*****=0.12**, ***P*****=0.000**	**1.02**
Education level	***B*****=** –**0.15,** ***P*** **<** **0.000**	***B*****=** –**0.16,** ***P*** **<** **0.000**	*B* = −0.07, *P* = 0.02	***B*****=** –**0.11,** ***P*** **<** **0.000**	*B* = −0.07, *P* = 0.02	1.14
Chronic diseases	*B* = 0.05, *P* = 0.05	*B* = 0.05, *P* = 0.08	*B* = 0.55, *P* = 0.07	*B* = 0.04, *P* = 0.18	***B*** **=** **0.09,** ***P*** **=** **0.002**	**1.04**
Psychiatric or psychological care	***B*** **=** **0.25,** ***P*** **<** **0.000**	***B*** **=** **0.24,** ***P*** **<** **0.000**	***B*** **=** **0.19,** ***P*** **<** **0.000**	***B*** **=** **0.19,** ***P*** **<** **0.000**	***B*** **=** **0.20,** ***P*** **<** **0.000**	**1.01**
*R^2^*	0.10	0.12	0.05	0.07	0.09	

*Factors included in the linear regression model are marked in bold. VIF, Variance Inflation Factor*.

## Discussion

Despite the limited evidence on the topic, we argue that it is time to discuss the relation between the level of knowledge about the pandemic and mental health, especially in the era of unlimited access and dissemination of unconfirmed information. In our study, we have confirmed the assumed hypothesis, demonstrating significant negative correlations between the level of knowledge about COVID-19 and the severity of psychopathological symptoms measured with the GHQ-28, both in relation to the total score and all its subscales, including somatic symptoms, severe depression, social dysfunction, anxiety and insomnia. However, using a linear regression model, we showed that only increased somatic symptoms are significantly associated with a lower level of knowledge about COVID-19.

In the study sample of over a thousand respondents, the majority had moderate level of knowledge about COVID-19 and the pandemic. This may suggest that the high availability and abundance of information in the media is not clearly reflected in the correctness of answers. This result corresponds to a study conducted from March 27 to April 15, 2020 in 15 countries worldwide, in which 51% of Poles declared moderate, and 39%—detailed knowledge about the symptoms of COVID-19 (55). However, it should be noted that in March the amount of research on the coronavirus or confirmed official information was much smaller than in the case of the second wave in September 2020. What is more, at that time, the media reported on an ongoing basis about the epidemiological situation of SARS-CoV-2, which significantly increased the level of anxiety in the general population (38). It is worth noting that the presented results refer to a specific segment of the population, which does not allow extrapolation of the results to the entire Polish population. In the study group, there was an overrepresentation of the female gender, higher education, city place of residence and (due to the nature of the study) access to the Internet. Such a group makes it possible to compare the obtained results with groups from other highly developed countries, while maintaining a similar socio-demographic structure. In the COVID-19 knowledge questionnaire more correct answers were provided by men, respondents with higher education and those not using psychiatric or psychological care. These results are consistent with the study by Zhong et al. (43) which also noted that greater knowledge is associated with male gender and higher education. It confirms the previous conclusions of Johnston et al. (39) about a causal link between education and health knowledge. The authors estimated that 1 year of education contributes to the increase of knowledge about health by up to 15%, measured with United Kingdom Health and Lifestyle Survey. The source of gaining knowledge is also important—approximately 60% of Internet users use online websites in order to look for the health-related information (56). According to Madden and Zickuhr (57), 65% of respondents use social networking sites, where the vast majority are women and young adults up to 30 years of age, and 69% of them do so every day. Narrowing the search for information to one channel, e.g., social media, may be associated with a lower level of knowledge about COVID-19, which results from reports describing the relationship between information sources and knowledge about the virus and pandemic (58). Moreover, women, compared to men, use social networking sites more often to obtain information on health-related issues 16. This would explain our results, as almost three-quarters of respondents were women, and time spent on social media increased significantly during the COVID-19 isolation (59). In our study, we did not observe a significant difference in the level of knowledge about COVID-19 between healthcare professionals and other professionals. This result can be considered in two ways: on the one hand, it may indicate a high level of knowledge in the society, which is comparable to that of healthcare professionals, and, on the other hand, it may indicate deficiencies in the education of those professionals. Longitudinal studies are needed to discern the direction of causation. Similarly, in terms of symptom severity as measured by the GHQ-28 scale, there was no significant difference between healthcare professionals and other respondents, which is a change from our study conducted during the first wave of the pandemic (24), in which medical professionals scored significantly higher in the total GHQ-28. This change can be explained by the phenomenon of habituation, as an ability to adapt to the new working conditions of healthcare workers. However, participants of the present study presented more severe psychopathological symptoms in comparison to the results of the study performed before the first wave of the COVID-19 pandemic assessing mental health of Polish nurses with the GHQ-28 questionnaire (60). The lack of significant differences between medical and non-medical professionals in terms of the level of knowledge about COVID-19 and the severity of psychopathological symptoms is consistent with the previously described correlation of knowledge about COVID-19 with the severity of psychopathological symptoms. Reported negative correlations between the level of knowledge about COVID-19 and the GHQ-28 score, both in relation to the total score and all its subscales indicate the necessity to consider two directions of causality. The first direction may point to the impact of insufficient knowledge about COVID-19 on the severity of psychopathological symptoms. This is confirmed by a study showing that better knowledge about the swine flu pandemic correlated with a reduction in the level of anxiety (61). In order to understand the mechanism of this cause-and-effect relationship, it is worth referring to reports assessing mental state in relation to information sources. A recently published study indicated that partially misleading and false news about COVID-19 generated higher levels of psychopathological symptoms, particularly anxiety (37, 62). In turn, according to Lin et al. (63), the use of social media was associated with the occurrence of insomnia, which was indirectly modified by the fear of COVID-19. Thus, it can be assumed that the limited amount of knowledge about the virus will imply greater anxiety and stress, which, with prolonged condition, causes a number of psychopathological symptoms. These, in turn, with less awareness of the disease, may be misinterpreted as symptoms of the SARS-CoV-2 itself. We also consider the second direction of causality, which involves the potential influence of the initial mental state on the knowledge of COVID-19. According to Pahayahay et al. (64), respondents with the highest levels of stress avoided the stressful tracking of COVID-19 reports that could potentially increase their knowledge. Depending on individual personality traits and strategies of coping with stress, individuals choose different sources of obtaining information and knowledge on health-related topics, and present an active or passive attitude (65). These differences may be explained by the use of various defense mechanisms. As a result of experiencing a high anxiety, our mind is likely to invoke fear-control responses like denial, which directly leads to avoidance and ignoring of information. According to Johnson (66) “ignoring happens when an individual consciously knows that a problem exists, but chooses not to confront it.” Further, mere ignorance inhibits information seeking, particularly in matters of health (67).

What is interesting, insufficient knowledge about COVID-19 pandemic showed weak but significant association with higher severity of somatic symptoms. Its relatively low value might be interpreted by the multiplicity and complexity of factors influencing the occurrence of psychopathological symptoms. Nevertheless, its importance should be emphasized. To date, to the best of our knowledge, this relationship has not been reported by other authors. However, focus on somatic symptoms, understood as the expression of the mental health, is widely described. According to Ran et al. (68), during the first wave of the COVID-19 pandemic, 45.9% of respondents showed somatic symptoms and in the general population of China, examined at two time points, their severity did not decrease over time compared to declining levels of perceived anxiety (69). Thus, our results give a new insight that higher reliable knowledge might decrease somatic symptoms. Moreover, referring to the conclusions from the first wave of the pandemic (24), according to medical professionals, prolonged emotional tension may lead to the development of somatic symptoms. From a psychodynamic perspective, such symptoms are understood as the effect of defense mechanisms—physical difficulties are more acceptable than depressive symptoms, which may lead to an occupational dysfunction. This hypothesis is in line with the results of study (70) that confirmed the independent influence of anxiety on the increased incidence of somatic problems, especially fatigue, pain, and gastrointestinal discomfort during the COVID-19 pandemic. Noteworthy, these symptoms often coincide with the course of COVID-19 itself. In an Italian study conducted during the general quarantine period in connection with the COVID-19 pandemic, a significantly higher incidence of somatic symptoms (headache, muscle pain, chills) was observed compared to similar studies (71). The most frequently reported symptoms were otolaryngological symptoms potentially related to COVID-19, such as cortex, cough, sore throat, and tinnitus. Presumably, somatic symptoms along with insufficient knowledge about the virus can be interpreted as symptoms of COVID-19 infection, which, according to the principle of positive feedback, may generate additional anxiety. Following this, people with reliable information about COVID-19 can more easily rationalize and distinguish the experienced somatic symptoms of anxiety from real symptoms of SARS-CoV-2. It is also concluded that the knowledge enables an objective estimation of the probability of infection and inhibits the vicious circle of the stress reaction resulting from the prolonged activation of the hypothalamic—pituitary—adrenal axis.

Our results should be interpreted also in context of the terms: Media Health Literacy and eHealth Literacy, which, according to the Nutbeam D. model (72) “are associated with health information seeking and health outcomes, such as health behavior and health status across various population groups.” However, these indicators were not measured in our study. Higher Media Health Literacy and eHealth Literacy help to better differentiate verified information from disinformation and fake news (73). Including these indicators in future studies could help to better understanding the impact of medical knowledge, including COVID-19, on the occurrence of psychopathological symptoms, and to develop programs to improve Media Health Literacy and eHealth Literacy in society.

## Limitations

The strength of our study is the first-time use of standardized questionnaires for measuring mental health and of original tool to assess the level of knowledge about COVID-19. However, we want to outline some of its limitations. First, the representativeness of the sample is limited due to the fact that the initial number of people who were asked to participate and the reasons for non-participation are not recorded. Another limitation of our study was the lack of the identification of the type of work of people outside the health service, which could allow for a better characterization of the study group. It should also be noted that in the study there were no questions regarding the duration of the selected symptoms, hence the results relate more to short-term experienced psychopathological symptoms than to long-term mental states. Inevitably, both the online distribution and the online form of the questionnaires themselves run the risk of bias in the responses, hence the strength of the evidence should be treated with caution. Moreover, we did not include information about the source of the respondents' knowledge about COVID-19, which could have been an additional advantage of the study in the light of the presented results. Finally, a significant limitation is the inability to establish a causal relationship between psychopathological symptoms and knowledge, hence we have attempted to present two directions of potential impact.

As a recommendation, we propose a moderate use of verified and diversified sources of information about the pandemic, due to the established relationship of knowledge about COVID-19 with the occurrence of symptoms of a stress reaction. It seems necessary to conduct further research on the relationship between mental health and the level and methods of searching for knowledge, also considering the sources of information obtained. Moreover, in view of the prolonged pandemic, longitudinal studies on representative samples are needed in order to make a reliable assessment of its long-term health and social consequences. Referring to the negative impact of information chaos, we take an unambiguous position on the essence of an objective and reliable presentation of the epidemiological situation by the media. In response to the growing phenomenon of infodemic, we believe it is rational to avoid sources of information presenting only sensational and disturbing information as well as sources underestimating the epidemiological threat. Consideration should be given to carrying out an information campaign aimed at improving the mental state of citizens by facilitating access to the necessary information. In our opinion, the role of WHO and other global organizations, which should have systematic access to the media in the event of a pandemic and global threat, is still fully unused and should prepare constantly updated information on safety, necessary pro-health behaviors, at the same time fully substantively and unambiguously refuting misleading views and beliefs born out of fear and ignorance.

## Conclusions

The results of our study confirm the initial hypothesis and indicate that the lower level of knowledge about the COVID-19 pandemic is associated with a greater severity of psychopathological symptoms. Therefore, we conclude that the complexity of the global pandemic problem makes it difficult to thoroughly search for information, thus downplaying the possible consequences of the problem and worsening the mental health. These findings highlight the new vital importance of objective and reliable media information on epidemiological issues. However, longitudinal studies are needed to discern the direction of causation.

## Data Availability Statement

The original contributions presented in the study are included in the article/Supplementary Material, further inquiries can be directed to the corresponding author/s.

## Ethics Statement

The studies involving human participants were reviewed and approved by Ethics Committee of the Medical University of Wroclaw (Poland, no 188/2020). The patients/participants provided their written informed consent to participate in this study.

## Author Contributions

JM, BM, DS, and JR: conceptualization. JM, BM, and DS: methodology. JM and BM: software. BS: validation. ML: formal analysis. JG and PG: investigation. JR: resources, supervision, and funding acquisition. MC and DŁ: data curation and visualization. JM, PG, JG, and DS: writing—original draft preparation. ML, BM, DS, and JR: writing—review and editing. JM: project administration. All authors have read and agreed to the published version of the manuscript.

## Conflict of Interest

The authors declare that the research was conducted in the absence of any commercial or financial relationships that could be construed as a potential conflict of interest.

## Publisher's Note

All claims expressed in this article are solely those of the authors and do not necessarily represent those of their affiliated organizations, or those of the publisher, the editors and the reviewers. Any product that may be evaluated in this article, or claim that may be made by its manufacturer, is not guaranteed or endorsed by the publisher.
